# Columbus Medical College

**Published:** 1882-09

**Authors:** 


### COLUMBUS MEDICAL COLLEGE.

Numerous pamphlets have been disseminated relative to the Columbus (Ohio) Medical College difficulties. The following extract from a recent number of the Maryland Med. Journal is a concise account of the affair, with some practical comments :

The revelations brought to light through the instrumentality of Dr. Jas. E. Reeves, of Wheeling, in reference to a medical college located in Columbus, Ohio, and known as the "Columbus Medical College," are so damaging to its reputation that we can hardly see how it can survive the odium into which it has been cast by them. One of the faculty, Dr. J. F. Baldwin, declared that "one man was graduated from this institution who didn't know what the iris was, nor the pupil ; could not locate the mitral nor tricuspid valves ; placed the valvulæ conniventes in the *brain*, and the ileo-cæcal valve in the *rectum!*"—adding, "there were several of that sort." For this exposure it appears he was summarily ejected from his professorship. This has led him to make further revelations, from which we learn that the leading spirit of this so-called college is a Dr. Hamilton, the professor of surgery, who owns the college building and a majority of the stock, so that he elects his own trustees, and through them causes himself to be elected treasurer and secretary of the board, and to be placed in charge of the building, and even of the dissecting material.

Dr. B. also states that diplomas have been granted after attending but one course, or a small part of one course, or even *without attending any course* at all ; that there are no hospital or clinical advantages except a surgical clinic once weekly, no museum worthy of the name, none but the crudest means of instruction, and only an illarranged college building. Yet this professes to be a *regular* college, and is a member of the American Medical College Association.

We have now in this city four Medical colleges, with the prospect of a fifth in the not distant future. There will necessarily be a keen rivalry between some of these, and the desire for large classes and success will prove a strong temptation to relax in the requirements relating to attendance, fees, and examinations. Let us be doubly careful that no just ground for censure attach to us. Better that the colleges should perish than that the honor and usefulness of the profession should be sacrificed.

May heaven defend us from ever witnessing in this community such things, or any approach to them, as have been brought to light in Columbus. May no rivalry, no supposed necessity, no engrossing self-interest, induce the authorities of *our* colleges to make any such sacrifices of decency, principle, and morality.—*Louisville Medical News.*

